# Retraction: Progesterone and calcitriol reduce invasive potential of endometrial cancer cells by targeting ARF6, NEDD9 and MT1-MMP

**DOI:** 10.18632/oncotarget.28760

**Published:** 2025-07-25

**Authors:** Sana Waheed, Batsukh Dorjbal, Chad A. Hamilton, G. Larry Maxwell, Gustavo C. Rodriguez, Viqar Syed

**Affiliations:** ^1^Uniformed Services University, Department of Obstetrics and Gynecology, Bethesda, MD 20814, USA; ^2^Women’s Health Integrated Research Center at Inova Health System, Department of Defense Gynecologic Cancer Center of Excellence, Annandale, VA 22003, USA; ^3^John P. Murtha Cancer Center at Water Reed National Military Medical Center, Bethesda, MD 20889, USA; ^4^Inova Fairfax Hospital, Department of Obstetrics and Gynecology, Falls Church, VA 22042, USA; ^5^Division of Gynecologic Oncology, North Shore University Health-System, University of Chicago, Evanston, IL 60201, USA; ^6^Uniformed Services University, Department of Molecular and Cell Biology, Bethesda, MD 20814, USA

**This article has been retracted:** This decision follows an investigation by Oncotarget journal regarding the concerns raised by the third party. Our image forensics analysis revealed several instances of internal and external overlaps and duplications. Specifically:

Figure 1: The IHC images of the negative controls (Grade III IgG) for ARF6 and NEDD9 show overlap.

Figure 3A: The b-actin WB for Ishikawa cells in siARF6 experiment was modified and reused for siNEDD9 experiment.

Figure 2: The B-actin blot is a modified duplicate of SOCS-3 Western blot in Figure 3D of an earlier unrelated paper [1].

Figure 7A: The Ishikawa cells Rho A WB is a duplicate of U937 cells cMyc WB in Figure 5B from 2015 unrelated paper [2].

Figure 7B: The HEC-1B cells N-WASP WB was reused from a 2016 paper by the same corresponding author [3], where it was presented as P21 expression in Ishikawa cells and simultaneously as MMP-9 in Figure 5B [3].

We also found that the WIP western blot in Figure 7A was later reused as TGF-b in Figure 2B in the 2019 paper of the same corresponding author [4], which has already been retracted.

Additionally, Uniformed Services University (USU), where the work was conducted, informed the journal about their internal research misconduct investigation. They found *“Reused and relabeled Ishikawa P21 lanes of Figure 3B in Oncotarget 2016; 7:69733-69749 as HEC-2B N-WASP (55 kDa) lanes in Figure 7B of Oncotarget 2017; 8:113583-113598” and requested the retraction of the paper. They also noted: “Although we have not received a response from Dr. Syed; she has not been an employee of the USU since January 2023, all other co-authors support this retraction request. Notably, no other co-author on the publication was implicated in the research misconduct investigation.”*

Considering both the USU findings and the results of our Scientific Integrity office investigation, the editorial decision has been made to retract the article. All authors and USU were informed of the retraction.

Original article: Oncotarget. 2017; 8:113583–113597. 113583-113597. https://doi.org/10.18632/oncotarget.22745
